# Supplementary material for: Target-directed microRNA degradation regulates developmental microRNA expression and embryonic growth in mammals
Source: bioRxiv. 2023 Jun 26:2023.06.26.546601. Preprint. [Version 1] doi: 10.1101/2023.06.26.546601 (PMC10327180; doi:10.1101/2023.06.26.546601)
Supplement: Supplement 1 [file media-1.pdf]

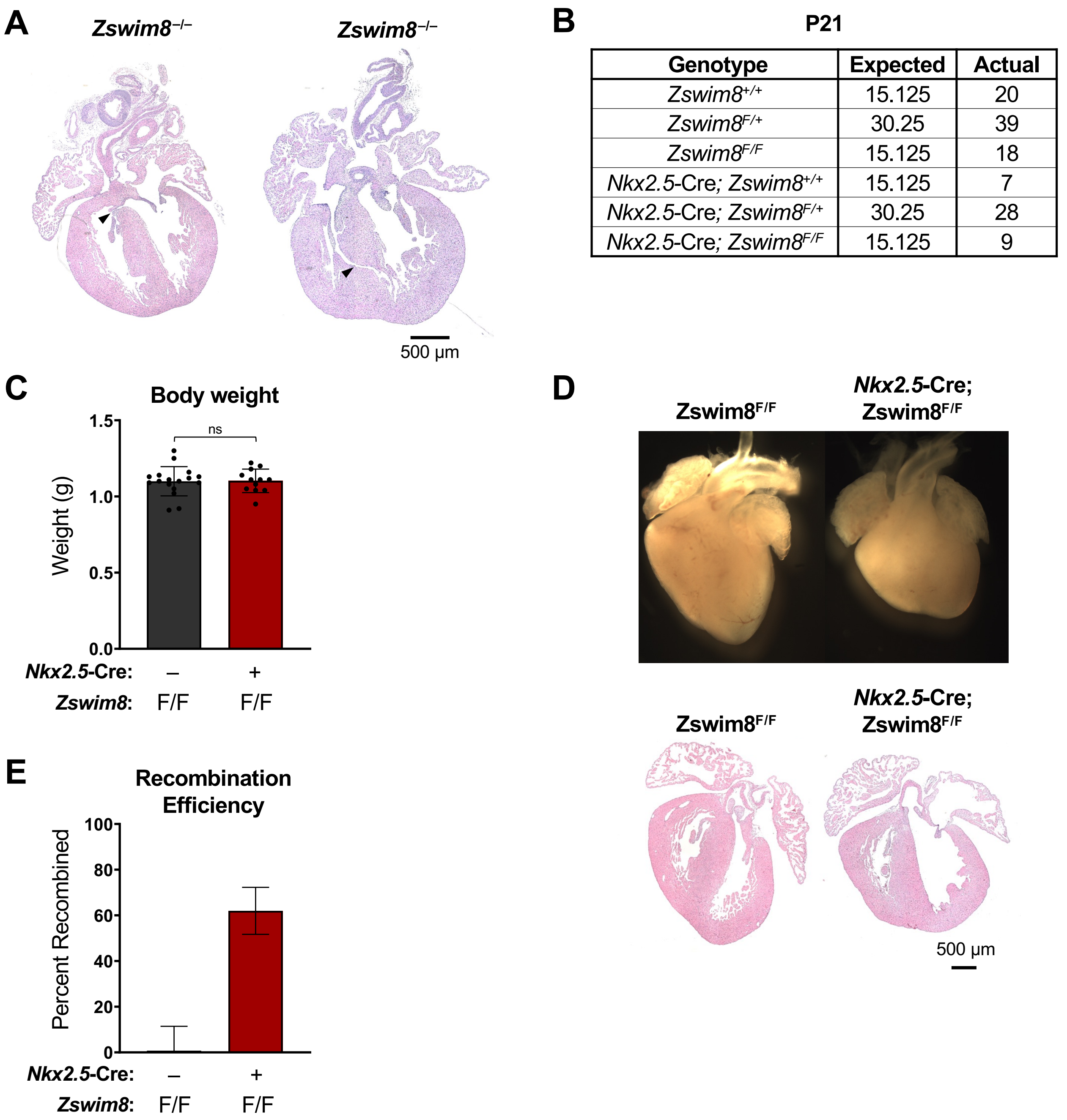

Genotype	Expected	Actual
Zswim8$^{+/+}$	15.125	20
Zswim8$^{F/+}$	30.25	39
Zswim8$^{F/F}$	15.125	18
Nkx2.5-Cre; *Zswim8*$^{+/+}$	15.125	7
Nkx2.5-Cre; *Zswim8*$^{F/+}$	30.25	28
Nkx2.5-Cre; *Zswim8*$^{F/F}$	15.125	9

Supplemental Figure S1. Analysis of mice with global or cardiac-specific loss of *Zswim8*. (*A*) Hearts from E18.5 *Zswim8*$^{–/–}$ mice showing presence of VSDs (arrowheads). (*B*) Frequency of genotypes of offspring at P21 produced from crossing *Nkx2.5*-Cre; *Zswim8*$^{+/F}$ to *Zswim8*$^{+/F}$ mice. (*C*) Body weights of E18.5 mice of the indicated genotypes. n=17 (*Zswim8*$^{F/F}$) and 12 (*Nkx2-5*^{Cre}; *Zswim8*$^{F/F}$). Data are represented as mean ± SD with individual data points shown. ns, not significant (unpaired *t*-test). (*D*) Images and H&E-stained sections of E18.5 hearts of the indicated genotypes. (*E*) Quantitative PCR measurement of *Zswim8*$^{F/F}$ allele recombination efficiency in E18.5 hearts from mice of the indicated genotypes. Data are represented as mean ± SD.

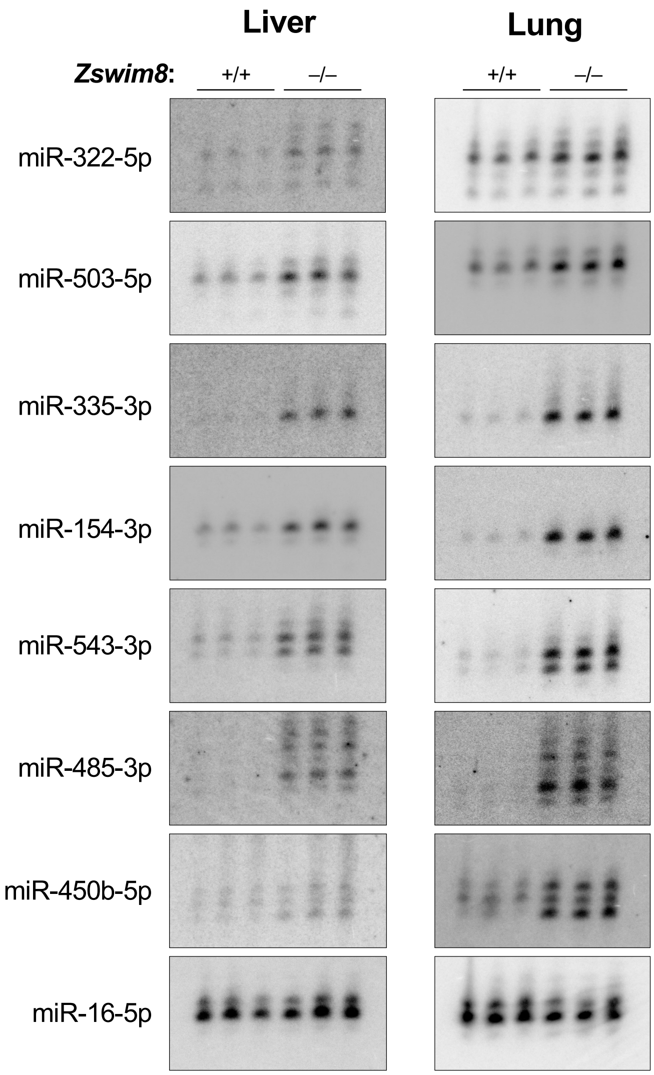

Supplemental Figure S2. **Northern blots of TDMD-regulated miRNAs in *Zswim8*$^{–/–}$ tissues.** Northern blot analysis of miRNA expression in E18.5 liver and lung from mice of the indicated genotypes. n=3 biological replicates per genotype. miR-16-5p served as a loading control.

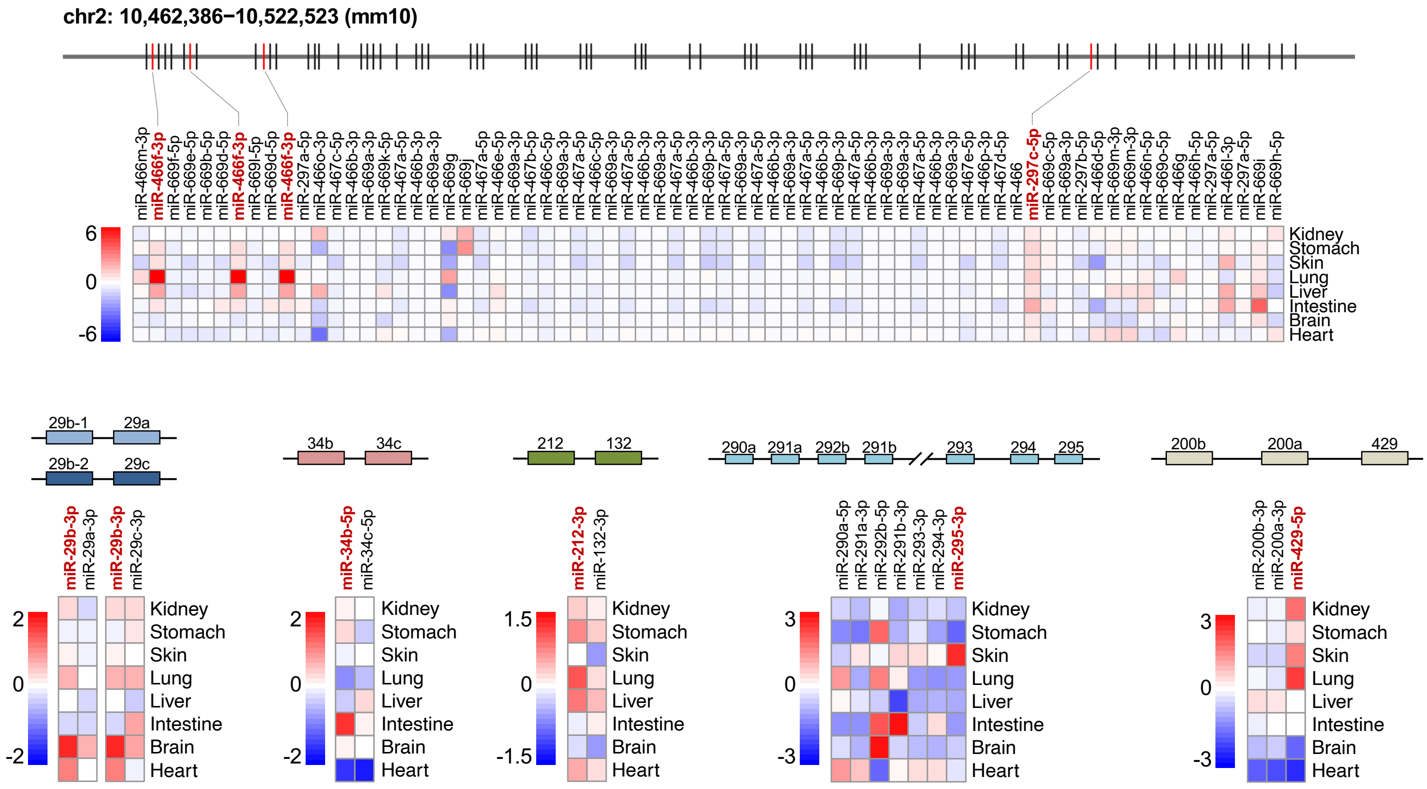

Supplemental Figure S3. **TDMD regulates clustered miRNAs.** Schematic representation of miRNA clusters encoding TDMD-regulated miRNAs. Heat maps display $\log_2$ fold change of miRNA expression (*Zswim8*$^{-/-}$/*Zswim8*$^{+/+}$) for each cluster member across tissues. miRNAs labeled in red text are TDMD substrates in at least one tissue.

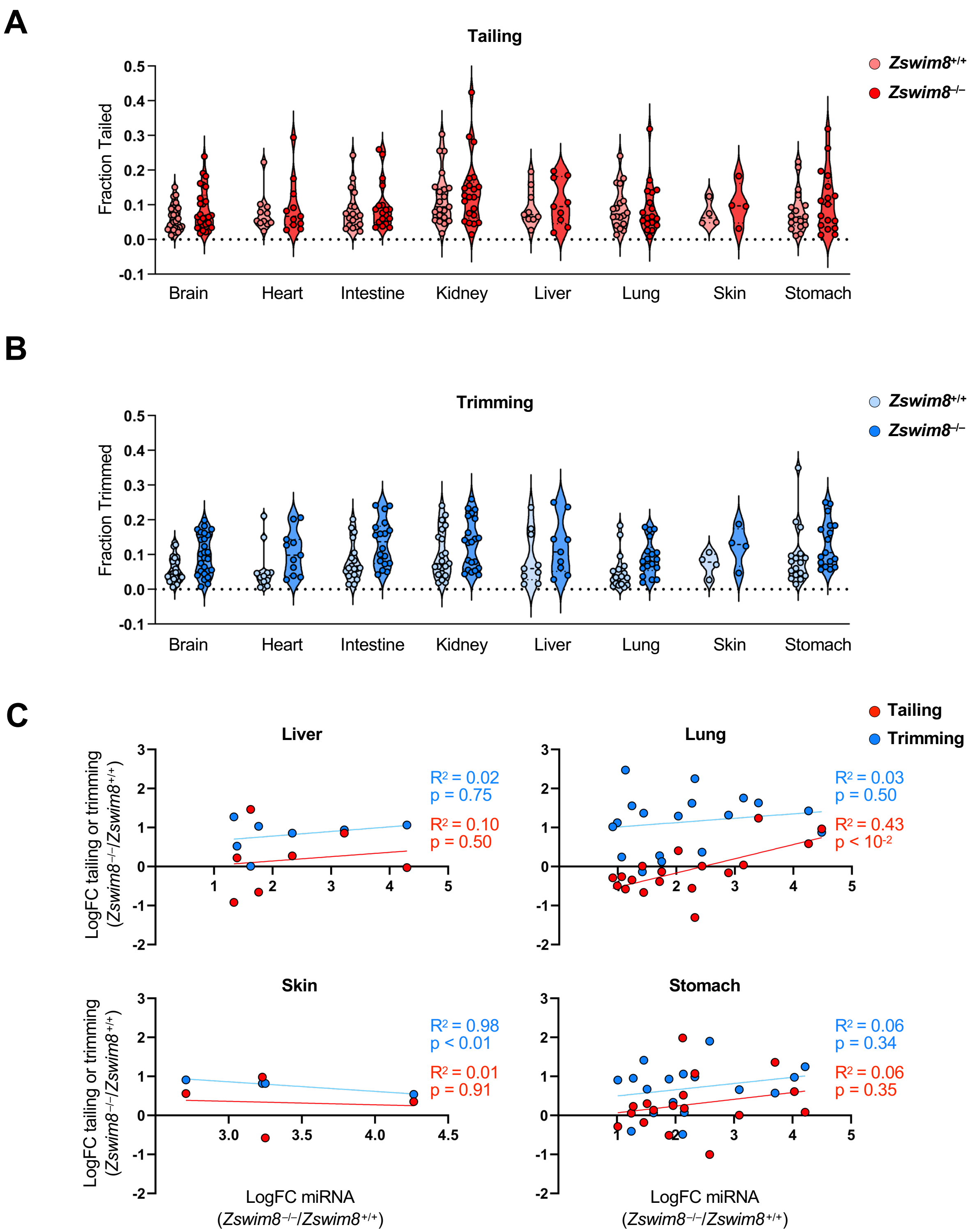

Supplemental Figure S4. Tailing and trimming of TDMD-regulated miRNAs. (*A*,*B*) Violin plots showing the fraction of tailed (*A*) or trimmed (*B*) isoforms of each TDMD-regulated miRNA in E18.5 tissues from *Zswim8*$^{+/+}$ or *Zswim8*$^{-/-}$ mice. (*C*) Scatter plots showing the fold change of tailing or trimming of each TDMD-regulated miRNA relative to its fold change in abundance (*Zswim8*$^{-/-}$/*Zswim8*$^{+/+}$) in each tissue.

A

P21 (male)

Zswim8	***miR-322/503***	**Expected**	**Actual**
+/+	+/Y	7.125	13
+/+	–/Y	7.125	8
+/–	+/Y	14.25	21
+/–	–/Y	14.25	15
–/–	+/Y	7.125	0
–/–	–/Y	7.125	0

B

P21 (female)

Zswim8	***miR-322/503***	**Expected**	**Actual**
+/+	+/–	8.375	16
+/+	–/–	8.375	13
+/–	+/–	16.75	20
+/–	–/–	16.75	18
–/–	+/–	8.375	0
–/–	–/–	8.375	0

C

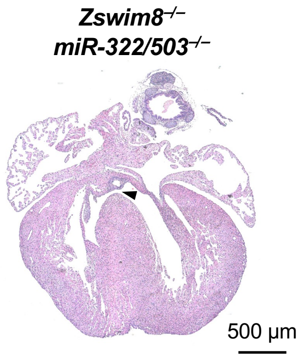

Supplemental Figure S5. **Loss of miR-322 and miR-503 does not rescue perinatal lethality or heart defects in *Zswim8*–/– mice.** (*A*,*B)* Frequency of genotypes of male (*A*) and female (*B*) offspring at P21 produced from intercrossing *Zswim8*+/–; *miR-322/503*+/– females to *Zswim8*+/–; *miR-322/503*–/Y males. (*C*) H&E-stained section of E18.5 *Zswim8*–/–; *miR-322/503*–/– heart mice showing presence of VSD (arrowhead).
